# Family Food Providers’ Perceptions of the Causes of Obesity and Effectiveness of Weight Control Strategies in Five Countries in the Asia Pacific Region: A Cross-Sectional Survey

**DOI:** 10.3390/nu9010078

**Published:** 2017-01-18

**Authors:** Anthony Worsley, Wei Wang, Rani Sarmugam, Quynh Pham, Judhiastuty Februhartanty, Stacey Ridley

**Affiliations:** 1 School of Exercise and Nutrition Sciences, Deakin University, Burwood 3125, Australia; w.wang@deakin.edu.au (W.W.); rs156@Uowmail.edu.au (R.S.); pth.quynh@hutech.edu.vn (Q.P.); s.ridley@deakin.edu.au (S.R.); 2 Health Promotion Board, 168937 Singapore, Singapore; 3 SEAMEO REFCON (Regional Centre for Food and Nutrition) Universitas Indonesia, Jakarta 10038, Indonesia; judhiastuty@yahoo.com

**Keywords:** obesity, perceptions, food providers, weight concerns, survey, Asia Pacific

## Abstract

The rise of the middle classes in developing countries and the associated epidemiological transition raises the importance of assessing this population group’s awareness of the causes of obesity and effective weight control strategies in order to develop effective health promotion strategies. The study aimed to examine the perceptions of the causes of obesity and weight control strategies held by middle class household food providers in Melbourne, Singapore, Shanghai, Indonesia and Vietnam. An online survey was conducted in late 2013, early 2014 among 3945 respondents. Information about body weight concerns, perceived causes of obesity, effectiveness of weight control methods, demographics, self-reported height and weight, and personal values was elicited. Confirmatory factor analyses (CFA) derived nine reliable factors which were used in structural equation modelling (SEM). Two thirds of respondents were trying to change their body weight, of them, 71% were trying to lose weight. The CFA and SEM showed that demographics, region of residence, personal values and perceptions of the causes of obesity (*Unhealthy food behaviours*, influences *Beyond personal control* and *Environmental influences*) had direct and indirect associations with three weight control methods factors, named: *Healthy habits, Eat less, sit less*, and *Dieting.* Middle class food providers in the study regions share public health views of obesity causation and personal weight control. These findings could inform public health and food policies, and the design of public health interventions and communications. Further research is required among lower socio economic status (SES) populations.

## 1. Introduction

Public health policies have been proposed to deal with the high prevalence of obesity, ranging from mass education programs to regulation of marketing and advertising [1,2]. The ultimate targets of these policies and programs are the general population, “consumers”. Understanding of the ways food consumers perceive the causes of obesity is needed to design communication programs and policies to foster less obesogenic habits. The implementation of successful policies depends on active consumer engagement [3,4,5]. It is important that policies are congruent with consumers’ opinions, for example, if consumers believe obesity is caused mainly by genetic factors, environmentally focused policies may be unsuccessful without detailed communication.

Since much of human physical activity and food intake is strongly influenced by the household environment [4], the household “gatekeeper” is an important type of food consumer. These individuals, often female, are responsible for food provision and transformation in the household; they influence the dietary behaviours of other household members [4]. Therefore, in this paper, we focus on the obesity-related interests of household food providers.

Generally, food providers’ views of the causes of obesity have been little studied. In our previous work, we showed that the Australian public held sophisticated views of the causes and prevention of children’s obesity [5]. These included several personal, environmental and genetic causes of childhood obesity such as overconsumption of unhealthy food and lack of healthy food and physical activity. We wanted to examine the ways in which members of the new influential middle classes in Eastern Asia [6,7,8] and present day Australians view the likely causes of obesity. We had several expectations about likely influences on these perceptions, as follows:

1. Demographic characteristics: Older people tend to be more concerned about food and health issues than younger people [9,10,11,12], as are most women [13]. Therefore, women and older people may view the causes of obesity differently to men and younger people. Highly educated people also tend to be more health conscious [14] and more able to afford exercise and dietary innovations [15], so they may emphasize individual responsibility for obesity. Similarly, being married may positively influence health and food consumption [16], and the presence of young children in the household may expose food providers to food marketing and obesogenic influences [17,18].
2. Body Mass Index status: People who are overweight or obese might be expected to have greater reason than others to consider the causes of obesity as well as methods to control body weight. In terms of attribution theory, their greater involvement suggests that as “actors” they may be more likely than slimmer people to attribute obesity to external causes beyond their personal control [19].
3. Personal value orientations: These are generally associated with attitudes and opinions [20]. For example, people with communitarian, “other-oriented” values [21] tend to look to external factors when explaining phenomena such as the role of government in the provision of school lunches [22], whereas those who hold more individualist values tend to place a greater emphasis on individual responsibility. We expected respondents with strong communitarian values [23] would emphasize the importance of external causes of obesity e.g., food marketing and environmental influences, and conversely, those with stronger self-oriented values would favour causes related to individual control, e.g., over-eating.
4. Levels of economic development: In regions undergoing rapid economic and epidemiologic transitions [24], the expanding middle class might be most exposed to consumerist (individualist) trends and fashions such as beauty culture, international film and media, and, food marketing. We hypothesized that people in economically developed regions (such as Melbourne and Singapore) would hold more individualistic views of obesity causation than those in recently developing regions (i.e., Indonesia, Vietnam and Shanghai). We expected household ownership of electronic communication and entertainment devices, indices of consumerist (individualist) culture, would parallel these regional differences.
5. Perceptions of effective weight control strategies: The perceived causes of obesity may influence perceptions of effective ways to control body weight. For example, those who believe environmental influences are mainly responsible for obesity might see environmental changes as the most effective ways to maintain a healthy body weight. Identification of food providers’ support for valid effective weight control activities could be used to develop supporting obesity prevention policies.

**Figure 1:**
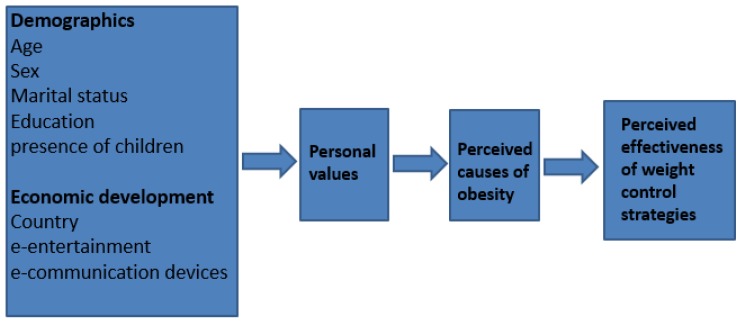
Initial conceptual model showing hypothesised pathways between demographic, country, personal values, perceived causes of obesity and perceived effectiveness of weight control strategies.

In summary, our aims were to examine the perceived causes of obesity among middle class people in five regions at different stages of economic development, and to examine their associations with demographics, body weight, personal values, economic development and perceptions of personal effective weight control methods. We proposed a conceptual model (Figure 1) based on the relative stability of predictors ranging from stable moderators like demographics, through less stable but enduring personal values and perceptions, to the more malleable body weight control practices (Figure 1). 

## 2. Materials and Methods

### 2.1. Design and Sampling

During December 2013 and January 2014, a detailed online survey (The Families and Food Survey) was conducted among approximately 800 household food providers in each of Indonesia, Melbourne, Shanghai, Singapore and Vietnam, by Global Market (GMI), an online market research company. This company maintains databases of volunteers who are rewarded by points for survey participation, the points being redeemable for small monetary payments. The main inclusion criteria were that respondents had to be household food providers, over 18 years and approximately 40 per cent were to be men to reflect possible changes in family food provisioning.

### 2.2. Procedure

Ethics permission for the study was granted by the Deakin University Health Ethics Advisory Group (HEAG 2013-163). The survey questionnaire was translated into Vietnamese, Bahasa Indonesia and Chinese by GMI. These translations were then checked by nationals of these countries (QP, JF, and WCW, respectively) and any culturally sensitive or misleading phrases were reworded. Prospective respondents were drawn from the GMI databases in the five regions. They were sent an email inviting them to take part in the survey.

### 2.3. The Questionnaire

The questionnaire included an extensive list of questions about household food issues. 

Responses to the following sets of questions are reported in this paper. 

• Self-reported height and weight

Respondents reported their height (in cm) and their weight (in kg) and these were converted into body mass indices [25]. BMI based on self-reported height and weight is a valid index of BMI for population studies [26].

• Demographic characteristics

Details were elicited of the respondents’ age, gender, marital status (single/widowed/separated/divorced, coded as 1, married/de facto as 2), and, highest level of education (completed primary school only and left school before 16 years of age, coded as 1, left school between 17 and 19 years, 2, trade or technical diploma, 3, university degree, 4). The number of children in the household was elicited for the 0–5, 5–12, 13–18 year age groups. In addition, the city or country of residence was dummy coded into four binary variables using Melbourne as the reference group.

• Household ownership of electronic communication and entertainment devices

Respondents were asked to indicate the numbers of electronic communication (e.g., smartphones, tablets, and computers) and entertainment devices (e.g., TVs, DVD players, games, etc.) in their households. The numbers of entertainment devices and communication devices that people owned were calculated (Table 1).

• Concern about body weight

Respondents were asked: *Are you concerned about your weight at the moment?* They could respond: Not at all (1); A little concerned (2); Somewhat concerned (3); or Very concerned (4).

Then they were asked: Are you trying to do anything about your weight? Are you trying to lose weight? and, Have you heard of the Body Mass Index (BMI)? They could answer: No (1); or Yes (2) to each of these questions (Table 2).

• Perceived causes of obesity

Thirteen items were taken from our previous study of the perceived causes of children’s obesity [5] (Table 3 and Supplementary Materials). Respondents were asked: *What do you think are the main causes of obesity?* In this and the next two sets of questions, five-point Likert scales were used (e.g., Not a cause of obesity, coded as 1, Not Sure/Neutral, 3, to Definitely a cause of obesity, 5). The order of items was rotated across respondents. It should be stressed that respondents were asked about the causes of obesity in general, not specifically about children’s or adult obesity.

• Perceived effectiveness of weight control strategies

Respondents were asked: *How effective are the following actions that individuals can take to maintain a healthy body weight?* A list of 23 possible actions was presented in rotated order (Table 4 and Supplementary Materials).

• Personal values

The 23-item Portrait of Values inventory [27] was administered. The original phrasing was altered to apply to both male and female respondents (Table 5 and Supplementary Materials). Respondents were asked: *How well do the following statements ACTUALLY describe you and your approach to life?*

• Analytical procedure

SPSS 22 [28] and Mplus 7.2 [29] were used for the data analyses. Confirmatory factor analyses (CFA) were conducted on the perceived causes of obesity, effective actions to maintain healthy body weight, and the personal values variables. This was done in order to identify latent (underlying) dimensions and to establish the construct validity of the items. Internal reliabilities (Cronbach alphas) were also reported. Structural equation modelling (SEM) was employed to test the hypotheses (further details in Supplementary Materials.) The dependent variables were the three composite variables derived from CFA of effective actions of maintaining healthy body weight namely, *Healthy habits, Eat less, sit less, and Dieting.* Individuals’ age, gender, education, marital status, children’s age ranges, number of electronic entertainment devices and communication devices), dummy coded regions of residence and BMI were used as independent variables. BMI was standardised with a mean of zero and standard deviation of 1.

## 3. Results

### 3.1. The Demographic Characteristics of the Samples

Fifty-seven per cent of the respondents were female; the mean age was 35.72 (SD 11.23) years (Table 1). Sixty per cent were married or in de facto relationships; 79% had at least a bachelor degree, home ownership and ownership of electronic communication and entertainment devices was high (Table 1).

### 3.2. Concern about Body Weight 

Fewer than half of the respondents were a little concerned or unconcerned about their body weight; 17.4% were very concerned. Greatest concern (“somewhat” plus “very”) was seen among respondents from Singapore and Vietnam (Table 2). Least concern was most prevalent among Indonesian respondents. Two thirds of respondents were trying to do something about their weight; of those, 71% were trying to lose weight, the highest proportions were found in Melbourne (88%) and Singapore (80%), the lowest in Vietnam (54%). Reported understanding of BMI was high (72%), highest in Singapore (91%) and Melbourne (84%) and lowest in Shanghai (55%).

### 3.3. Perceived Causes of Obesity

There was much agreement between the respondents in the different countries although there were some major differences (Table 3). Item comparisons associated with the three reliable factors derived from CFA are described below. 

*Unhealthy food behaviour:* About 80% indicated that overconsumption of sugar sweetened beverages (SSB), eating oversized servings of food, and regular consumption of fast foods were causes of obesity, though respondents in Vietnam rated these (and other causes) lower than respondents in other countries. 

*Beyond personal control:* “Lack of awareness of the dangers of obesity” was cited most by Indonesians and least by Melbournians. “Poor availability of healthy foods” was indicated least by the Vietnamese respondents. “Lack of physical activity opportunities” was indicated most by people in Shanghai and least by Melbourne respondents. Genetic causation of obesity was most endorsed by Singaporeans and least by Vietnamese.

*Environmental influences:* Modern technology and the promotion of unhealthy foods was blamed most by respondents in Melbourne, Singapore and Shanghai, and least by those in Indonesia and Vietnam. The low cost of unhealthy foods was endorsed least by the Vietnamese and most by the Melbournians. 

### 3.4. Perceived Effectiveness of Personal Weight Control Options

CFA revealed three factors relating to effective actions to maintain healthy BMI: *Healthy habits, Eat less, sit less* and *Dieting* (Table 4). Differences among the item responses were found across countries.

*Healthy habits*: “Establish an exercise routine” was supported most by Indonesian and least by Vietnamese respondents. “Make a regular shopping list”, and, “Try not to eat sweetened foods like cakes or confectionery” were endorsed most by Melbournians and least by Vietnamese. “Avoid alcoholic drinks” was supported most by Indonesian and least by Shanghai respondents. 

*Eat less, sit less*: “Don’t sit down for longer than 15–20 min at a time” was endorsed most by the Shanghainese and least by Indonesians. “Use smaller plates and dishes”, “Don’t have second helpings”, and “Try to eat less” were supported most by Melbournians and least by Vietnamese.

*Dieting:* “Go on a slimming diet” and “Substitute diet soft drinks for regular soft drinks” were endorsed most by Vietnamese respondents and least by Melbournians. “Weigh yourself regularly” and “Use commercial meal replacements” were supported most by Indonesians and Shanghainese, respectively, and least by Melbournians.

The CFA of the personal values ratings yielded three reliable factors, provisionally labelled as Conformity-security-tradition, Self-oriented-hedonism, and Equality-nature (Table 5).

### 3.5. SEM Pathways 

**Figure 2:**
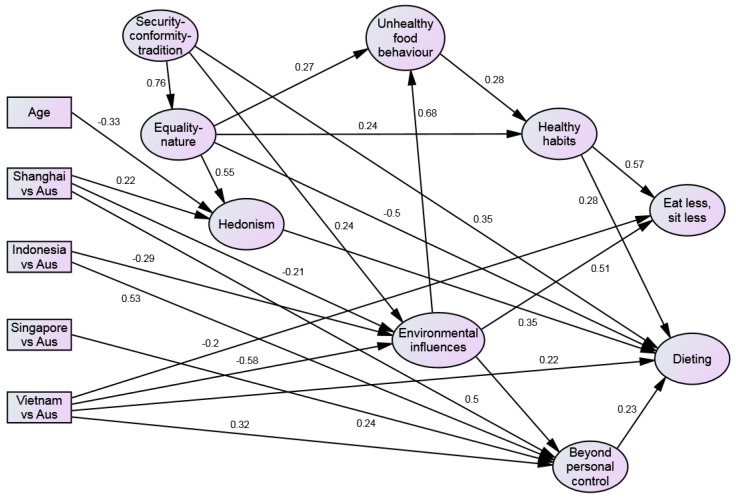
Structural equation model with the standardised pathway coefficients greater than 0.20 being displayed. Note: only regression weights >0.20 are shown here.

Figure 2 illustrates the path model with the unstandardized parameter estimates. 

*Eat less, sit less* was positively related to *Healthy habits* (β = 0.74, *p* < 0.01), *Security-conformity-tradition* (β = 0.21, *p* < 0.01), age (β = 0.01, *p* < 0.01), female gender (β = 0.27, *p* < 0.01), number of e-entertainment devices owned (β = 0.09, *p* < 0.01), and *Environmental influences* (β = 0.50, *p* < 0.01) but negatively linked to being Indonesian (β = −0.38, *p* < 0.01), Singaporean (β = −0.29, *p* < 0.01), or Vietnamese (β = −0.50, *p* < 0.01) (vs. Melbournian). 

*Dieting* was positively associated with Healthy habits (β = 0.42, *p* < 0.01), *Security-conformity-tradition* (β = 0.34, *p* < 0.01), *Self-oriented hedonism* (β = 0.35, *p* < 0.01), being Indonesian (β = 0.27, *p* < 0.01) or Vietnamese (β = 0.54, *p* < 0.01) (vs. Melbournian), and *Beyond personal control* (β = 0.23, *p* < 0.01) but negatively related to *Equality-nature* (β = −0.50, *p* < 0.01). 

*Healthy habits* were positively associated with *Unhealthy food behaviour* (β = 0.18, *p* < 0.01), female gender (β = 0.08, *p* < 0.01), *Equality-nature* (β = 0.16, *p* < 0.01), being Vietnamese (β = 0.24, *p* < 0.01) (vs. Melbournian) and *Environmental influences* (β = 0.12, *p* < 0.01) but negatively related to being Singaporean (β = −0.06, *p* < 0.01) (vs. Melbournian).

*Unhealthy food behaviour* was directly and positively related to female gender (β = 0.20, *p* < 0.01), *Equality-nature* (β = 0.28, *p* < 0.01), being Indonesian (β = 0.34, *p* < 0.01) (vs. Melbournian), and *Environmental influences* (β = 0.68, *p* < 0.01) but negatively associated with number of children aged between 0 and 5 years (β = −0.06, *p* < 0.01) *and Self-oriented-hedonism* (β = −0.19, *p* < 0.01). 

*Beyond personal control* was positively associated with *Security-conformity-tradition* (β = 0.15, *p* < 0.01), being Shanghainese (β = 1.25, *p* < 0.01), Indonesian (β = 1.33, *p* < 0.01), Singaporean (β = 0.61, *p* < 0.01), or Vietnamese (β = 0.80, *p* < 0.01) (vs. Melbournian), and *Environmental influences* (β = 0.78, *p* < 0.01).

*Environmental influences* were positively linked to *Security-conformity-tradition* (β = 0.24, *p* < 0.01) and education (β = 0.03, *p* < 0.05) but negatively related to being Shanghainese (β = −0.51, *p* < 0.01), Indonesian (β = −0.74, *p* < 0.01), or Vietnamese (β = −1.44, *p* < 0.01) (vs. Melbournian).

On a bivariate level, the standardized BMI was associated with *Unhealthy food behaviour* (*r* = 0.10, *p* < 0.01), *Environmental influences* (*r* = 0.14, *p* < 0.01), and *Eat less, sit less* (*r* = 0.19, *p* < 0.01) and related to *Beyond personal control* (*r* = 0.04, *p* < 0.05), and *Healthy habits* (*r* = 0.04, *p* < 0.05). However, these significant bivariate relationships are not evident in the multivariate model.

## 4. Discussion

It is clear from the high levels of home ownership, education and ownership of electronic devices that most of the respondents can be regarded as “middle-class” with considerable purchasing power. Our findings are complex but there are at least five clear themes which have relevance for obesity prevention in the Asia Pacific region. 

First, there were many similarities between the regions. Concern with body weight and interest in losing weight was high in these middle class respondents. This is similar to survey findings from the US, the UK and other “Western” countries (e.g., International Food Information Council surveys [30]). The most commonly perceived causes of obesity related to unhealthy food behaviour and lack of physical activity. Similarly, the most effective strategies to control weight related to establishment of exercise regimens and avoidance of sweet foods, alcohol and second helpings, and, use of shopping lists. This commonality suggests that public health authorities in these regions can learn from each other about ways to influence population awareness and health habits. Many of these popular views of obesity causation and weight control effectiveness are substantiated in the research literature (e.g., the effects of sweet foods and beverages [31,32]), alcohol reduction and regular exercise on body weight). The apparent validity of these popular views suggests that they might be incorporated into the food and health policies of the respondents’ countries. 

Second, there were also major national differences. These tended to be consistent, with exceptions, with our economic development hypothesis. Respondents in developed economies, Melbournians and Singaporeans tended to share similar views of many topics, and those in developing economies, particularly the Vietnamese and Shanghainese respondents, held differing views. However, other cultural factors appear to have influenced the findings such as the greater opposition to the promotion of alcoholic drinks in Indonesia which has a large Muslim population. The Vietnamese respondents understated most of the perceived causes of obesity. This may be because obesity is a new phenomenon in Vietnam and people have had little time to become familiar with it. Lack of awareness of obesity was reported most by Indonesian respondents and least by Melbournians. The divide between Melbourne and the other regions is most apparent in the SEM (Figure 2). There were major differences shown by large path coefficients between Melbourne and the other regions on the *Beyond personal control factor* (Melbournians scoring lower than the others) but no difference was seen between Melbournians and Singaporeans on the *Environmental influence* factor. In contrast the Melbournians scored more highly on *Environmental influences* than Indonesian, Shanghainese and Vietnamese respondents. This may be related to the media coverage of industry’s role in the causation of obesity in Australia. There was only partial support for the role of e-entertainment and computer ownership which were weakly associated with *Self-oriented hedonism* and *Equality-nature* and thence with several perceived causal and effectiveness factors.

Third, causal perceptions predicted effectiveness strategies, e.g., *Environmental influences* was negatively associated with *Unhealthy food behaviour*, positively with *Healthy habits* and *Eat less, sit less*. Since causal perceptions are likely to be malleable, they provide the opportunity for awareness raising communications to bring about changes in the perceived effectiveness of a number of weight control strategies thus affecting readiness to change. Individual outcome factors like *Dieting* can be examined to determine their aetiology to identify foci for communication programs.

Fourth, the three derived values factors are similar to the three main segments postulated in Schwartz’s values taxonomy [23]. Their relationships with the perceived causal and effectiveness factors were generally as hypothesised. In particular, *Security-conformity-tradition* and *Equality-nature* played major mediating roles between the stable demographic factors and the perceptual factors. From a health promotion perspective, they could be used to segment the respondents to identify perceptual and other characteristics which could be the foci of communication programs [33]. In further research the hypothesis that “other oriented” people (with strong *Security- conformity-tradition* and *Equality-nature values*) resist the adoption of consumerist practices like soft drink consumption could be tested. Certainly the interests of these people in the common good should be supported by health and food policies. Finally, as expected, Self-oriented Hedonism was associated with lesser emphasis on *Unhealthy food behaviour* causes and more with *Dieting* as an effective form of weight control.

Fifth, demographics played only small roles in the SEM. However, the findings were consistent with earlier research [10,11,12,13,14,15,16,17]. For example, older age and female gender were directly and positively associated with *Healthy habits* and *Eat less*, *sit less*. More novel was the positive relationship between higher education and *Environmental influences* and the weak, negative relationship between *Unhealthy food behaviour* and the presence of children under 5 years. Food providers with children in this age group tended to dismiss the consumption of oversized servings of foods, sugar sweetened drinks and fast food as causes of obesity. This requires further confirmation and explanation but it suggests that public health messages about these products may need to be communicated more effectively to parents and caregivers. The other relationships with causal and effectiveness factors were mediated by the personal values factors. Finally, contrary to our expectations, food providers’ BMI was unrelated to any of the examined factors in the SEM, even though BMI was significantly correlated with these factors at the bivariate level. That is, these particular obesity beliefs appeared to be independent of weight status.

We have shown in this paper that members of the middle class in the five countries have sophisticated views about the causes and individual responses to overweight and obesity. The question that arises is whether the public health policies in these countries are congruent with the respondents’ views? In developed countries such as Australia and Singapore, obesity and non-communicable diseases have been on public health agenda for many years and many health promotion activities have been designed to address the issue. In contrast, Indonesia, Vietnam, and China, are still dealing with under nutrition and stunting whilst also experiencing rapid increases in obesity and non-communicable diseases, particularly in urban areas. This double burden of disease has been well described in the nutrition literature [24]. However, there are relatively few resources in developing economies to adopt whole of population approaches to the prevention of obesity as well as under nutrition. Hopefully, as economies develop there will be more resources available for the implementation of anti-obesogenic policies.

### Limitations 

This exploratory study has several limitations. First, although the online survey administration was a good way to reach middle class respondents, the final samples are highly skewed to middle class, highly educated, well off respondents. It is possible that the high purchasing power of the respondents may enable them to purchase healthier, less energy dense foods than people from less well off backgrounds [34] thus influencing their attitudes to obesity. Future research should also include people from lower socio economic strata in order to assess the beliefs of the whole population. Similarly, the samples included only people who were household food providers. It is likely that non-food providers have similar views but this needs to be established in future research. There may be important beliefs held by members of the community that we have not assessed; the inclusion of open ended probe questions in future studies may be an informative improvement. 

## 5. Conclusions

The findings show that middle class food providers in the Asia Pacific Region are concerned with body weight and share public health advocates’ views about the causes of obesity and personal ways to control body weight. The findings can inform public health and food policies, and the identification of mediating values and perceptual variables could aid the design of health interventions and communications in the region. Further research is required among lower SES populations.

## Figures and Tables

**Table 1:** The demographic characteristics, BMI and ownership of communication devices of the samples.

	Melbourne *n* = 769	Shanghai *n*= 807	Indonesia *n* = 788	Singapore *n* = 771	Vietnam *n* = 810	Total *n* = 3945
Gender: Female (%)	58.4	57.2	59.5	49.3	60.5	57.3
* Age: Mean (Std Dev)	41.43 (12.70)	37.81 (10.53)	32.97 (9.07)	37.45 (11.68)	29.25 (7.35)	35.72 (11.23)
Marital status: Married/de facto (%)	61.0	77.4	57.2	55.3	51.4	60.4
Education: Bachelor degree or higher (%)	58.9	89.5	80.8	74.2	90.7	78.8
Families with children between 0 and 5 years (%)	20.4	34.9	39.3	25.9	54.2	35.2
Families with children between 6 and 12 (%)	18.1	16.5	30.9	21.4	27.8	23
Families with children between 13 and 18 (%)	15.6	14.7	25.1	19.2	21.4	19.2
BMI: Mean (Std Dev)	26.89 (7.03)	23.60 (6.92)	23.11 (5.17)	23.08 (4.53)	20.81 (3.32)	23.47 (5.91)
Own or buying household accommodation (%)	65.8	89.2	67.1	85.1	73.8	76.3
E-communication devices: Mean (Std Dev)	3.7 (2.1)	4.2 (1.6)	3.9 (2.4)	5.0 (2.4)	3.8 (2.0)	4.1 (2.2)
E-entertainment devices: Mean (Std Dev)	5.7 (3.3)	4.9 (2.0)	4.7 (2.8)	5.1 (2.8)	4.3 (2.8)	4.9 (2.8)

* Range: 18–64 years.

**Table 2:** Household food providers’ body weight concerns and weight change views and practices across study regions (%).

		Melbourne (*n* = 769)	Shanghai (*n* = 807)	Indonesia (*n* = 788)	Singapore (*n* = 771)	Vietnam (*n* = 810)	Total (*n* = 3945)	Chi-square	*p*-Value
Are you concerned about your weight at the moment?	Not at all	20.6	22.8	26.6	14.7	14.4	19.8	170.70	<0.01
	A little concerned	29.5	28.0	28.8	19.3	21.7	25.5		
	Somewhat concerned	33.0	38.7	32.5	38.8	43.2	37.3		
	Very concerned	16.9	10.5	12.1	27.2	20.6	17.4		
Are you trying to do anything about your weight?	Yes	61.7	67.3	72.5	61.6	70.7	66.8	36.29	<0.01
* Are you trying to lose weight?	Yes	53.9	42.5	53.8	49.0	37.9	47.3	182.63	<0.01
* Are you trying to gain weight?	Yes	3.2	4.5	13.5	6.1	26.0	10.8	197.82	<0.01
* Are you trying to maintain weight?	Yes	3.5	17.5	4.5	4.7	5.2	7.1	7.84	>0.05
Have you heard of the Body Mass Index (BMI)?	Yes	83.9	55.0	60.7	91.4	69.4	71.8	379.08	<0.01

**Table 3:** Household food providers’ views of the causes of obesity (per cent definite cause, ratings 4 + 5).

	Melbourne (*n* = 769)	Shanghai (*n* = 807)	Indonesia (*n* = 788)	Singapore (*n* = 771)	Vietnam (*n* = 810)	Total (*n* = 3945)	Chi-square	*p*-Value
*Unhealthy food behaviour* (α = 0.68)								
Eating oversized servings of foods	87.8	88.7	89.3	84.0	69.4	83.8	300.48	<0.01
Regular consumption of fast foods	89.6	79.7	79.6	87.0	70.9	81.2	270.07	<0.01
Overconsumption of sugar sweetened drinks	88.9	86.1	89.1	86.6	74.2	84.9	257.85	<0.01
*Beyond personal control* (α = 0.55)								
People aren’t aware of the dangers of obesity	56.8	67.9	81.7	65.5	65.6	67.6	223.38	<0.01
Lack of availability of healthy foods	41.0	65.4	53.8	50.3	32.7	48.7	273.02	<0.01
Lack of physical activity opportunities	66.6	87.0	81.6	76.9	70.5	76.6	238.76	<0.01
Genes cause obesity	44.5	61.8	58.4	62.4	36.5	52.7	387.60	<0.01
*Environmental influences* (α = 0.59)								
Modern technology (e.g., cars, computers, video games)	68.8	62.3	43.3	64.9	45.9	56.9	343.08	<0.01
The promotion of unhealthy foods (in stores, the mass media and online)	72.8	64.9	55.3	73.4	35.7	60.2	438.28	<0.01
The low cost of unhealthy food	71.4	41.3	57.1	62.1	30.1	52.1	400.97	<0.01

Note: Not all items loaded on the confirmatory factors, see Supplementary Materials.

**Table 4:** Household food providers’ perceptions of the views of the effectiveness of ways to maintain body weight (per cent effective, ratings 4 + 5).

	Melbourne (*n* = 769)	Shanghai (*n* = 807)	Indonesia (*n* = 788)	Singapore (*n* = 771)	Vietnam (*n* = 810)	Total (*n* = 3945)	Chi-square (*df = 8*)	*p*-Value
*Healthy habits* (α = 0.65)								
Establish an exercise routine	87.7	87.5	91.9	84.8	84.7	87.4	42.29	<0.01
Make a regular shopping list	65.1	57.9	55.0	50.6	61.4	58.0	68.69	<0.01
Try not to eat sweetened foods like cakes or confectionery	75.7	67.9	68.6	69.9	64.4	69.2	38.78	<0.01
Avoid alcoholic drinks	58.1	49.4	77.6	59.9	56.4	60.2	167.38	<0.01
*Eat less, sit less* (α = 0.70)								
Don’t sit down for longer than 15–20 min at a time	43.8	68.9	40.0	42.0	47.7	48.6	223.41	<0.01
Use smaller plates and dishes	68.8	55.8	36.8	54.6	30.1	49.0	470.46	<0.01
Don’t have second helpings	72.5	58.9	66.2	59.1	31.2	57.4	517.43	<0.01
Try to eat less	70.4	51.1	54.4	58.2	32.5	53.1	428.91	<0.01
*Dieting* (α = 0.68)								
Go on a slimming diet	30.4	41.0	50.4	30.9	57.3	42.2	202.22	<0.01
Weigh yourself regularly	39.0	47.5	56.6	52.0	53.8	49.8	87.37	<0.01
Substitute diet soft drinks for regular soft drinks	23.5	31.7	46.9	29.3	62.6	39.0	425.78	<0.01
Use commercial meal replacements	19.0	31.2	29.5	21.9	30.5	26.5	138.06	<0.01

Note: not all the items are represented on the confirmatory factors, see Supplementary Materials for the complete list.

**Table 5:** Household food providers’ personal values (per cent like me, ratings 4 + 5).

	Melbourne (*n* = 769)	Shanghai (*n* = 807)	Indonesia (*n* = 788)	Singapore (*n* = 771)	Vietnam (*n* = 810)	Total (*n* = 3945)	Chi-square (*df = 8*)	*p*-Value
*Security-Conformity-Tradition* (α = 0.68)								
1. I prefer to live in secure surroundings and avoid doing things that might endanger my safety.	61.7	63.3	69.5	59.1	71.5	65.1	48.41	<0.01
2. I always try to follow the rules of society and do what is expected of me, even when no one is watching.	54.7	54.4	62.0	48.6	56.2	55.2	37.10	<0.01
3. I always try to behave properly and to avoid doing anything people would say is wrong.	57.1	44.1	61.9	52.0	68.9	56.8	135.73	<0.01
4. I adhere to traditions and try to follow the customs handed down to me by religion or family.	43.5	58.5	64.6	44.0	53.1	52.8	164.33	<0.01
*Self-oriented Hedonism* (α = 0.73)								
1. I like to be constantly surprised, to do many different things in my life, and always look for new things to do.	39.1	48.2	55.1	44.0	52.3	47.8	73.54	<0.01
2. I like being seen as very successful and recognised by others for my achievements.	27.4	59.2	37.1	43.1	48.4	43.2	310.13	<0.01
3. I always seek adventure and take risks to lead an exciting life.	25.1	30.2	41.7	36.3	39.0	34.5	152.56	<0.01
4. I take every chance I have to seek out fun and to always do the things that give me pleasure.	35.1	63.9	47.5	42.0	70.4	52.1	334.35	<0.01
*Equality-nature* (α = 0.74)								
1. I believe it’s important that every person in the world should be treated equally no matter who, where, or what they are.	73.5	70.8	81.0	66.1	73.1	72.9	58.83	<0.01
2. I believe everyone should have equal opportunities in life no matter who, where, or what they are.	76.6	74.1	81.7	68.7	76.2	75.5	38.89	<0.01
3. I am always willing to listen to people who are different and even when I disagree with them I still want to understand them.	62.7	59.9	71.6	55.0	71.4	64.2	76.48	<0.01
4. I care for nature and always look after the Environmental influences.	59.9	71.6	71.6	54.6	58.1	63.2	105.99	<0.01

Note: the full list of personal values items is given in the Supplementary Materials.

## Supplementary Materials

The following are available online at http://www.mdpi.com/2072-6643/9/1/78/s1, Table S1: Food Providers’ views of the causes of obesity, Table S2: Food Providers’ perceptions of the views of the effectiveness of ways to maintain body weight, Table S3: Adaption of Portrait of Values, Structural Equation Model: Analytical Procedure.
