# Supplementary material for: Family Food Providers’ Perceptions of the Causes of Obesity and Effectiveness of Weight Control Strategies in Five Countries in the Asia Pacific Region: A Cross-Sectional Survey
Source: Nutrients. 2017 Jan 18;9(1):78. doi: 10.3390/nu9010078 (PMC5295122; doi:10.3390/nu9010078)
Supplement: Supplementary file 1 [file nutrients-09-00078-s001.docx]

Supplementary Materials: Family Food Providers’ Perceptions of the Causes of Obesity and Effectiveness of Weight Control Strategies in Five Countries in the Asia Pacific Region: A Cross-Sectional Survey

Anthony Worsley, Wei Wang, Rani Sarmugam, Quynh Pham, Judhiastuty Februhartanty and Stacey Ridley

**Table S1.** Food Providers’ views of the causes of obesity (% definite cause, ratings 4 + 5).

|  | **Melbourne  *n* = 769** | **Shanghai  *n* = 807** | **Indonesia  *n* = 788** | **Singapore  *n* = 771** | **Vietnam  *n* = 810** | **Total  *n* = 3945** | **Chi Sq** | ***p*** |
| --- | --- | --- | --- | --- | --- | --- | --- | --- |
| Overconsumption of sugar sweetened drinks | 88.9 | 86.1 | 89.1 | 86.6 | 74.2 | 84.9 | 257.845 | <0.001 |
| People don’t do enough physical activity | 89.5 | 88.7 | 89.6 | 82.9 | 70.4 | 84.1 | 395.081 | <0.001 |
| Eating oversized servings of foods | 87.8 | 88.7 | 89.3 | 84.0 | 69.4 | 83.8 | 300.484 | <0.001 |
| Regular consumption of fast foods | 89.6 | 79.7 | 79.6 | 87.0 | 70.9 | 81.2 | 270.073 | <0.001 |
| Lack of physical activity opportunities | 66.6 | 87.0 | 81.6 | 76.9 | 70.5 | 76.6 | 238.756 | <0.001 |
| People aren’t aware of the dangers of obesity | 56.8 | 67.9 | 81.7 | 65.5 | 65.6 | 67.6 | 223.379 | <0.001 |
| Lack of willpower | 72.7 | 69.5 | 61.2 | 73.8 | 54.8 | 66.3 | 315.363 | <0.001 |
| The promotion of unhealthy foods (in stores, the mass media and online) | 72.8 | 64.9 | 55.3 | 73.4 | 35.7 | 60.2 | 438.277 | <0.001 |
| Modern technology (e.g., cars, computers, video games) | 68.8 | 62.3 | 43.3 | 64.9 | 45.9 | 56.9 | 343.078 | <0.001 |
| Genes cause obesity | 44.5 | 61.8 | 58.4 | 62.4 | 36.5 | 52.7 | 387.597 | <0.001 |
| The low cost of unhealthy food | 71.4 | 41.3 | 57.1 | 62.1 | 30.1 | 52.1 | 400.970 | <0.001 |
| Lack of availability of healthy foods | 41.0 | 65.4 | 53.8 | 50.3 | 32.7 | 48.7 | 273.021 | <0.001 |
| Lack of safe cycling and walking paths | 31.5 | 21.1 | 34.9 | 43.3 | 27.7 | 31.6 | 132.652 | <0.001 |

**Table S2.** Food Providers’ perceptions of the views of the effectiveness of ways to maintain body weight (% Effective, ratings 4 + 5).

|  | **% Melbourne  *n* = 769** | **% Shanghai  *n* = 807** | **% Indonesia *n* = 788** | **% Singapore  *n* = 771** | **% Vietnam  *n* = 810** | **% Total  *n* = 3945** | **Chi Sq.** | ***p*** |
| --- | --- | --- | --- | --- | --- | --- | --- | --- |
| Establish an exercise routine | 87.6 | 87.5 | 92.1 | 84.8 | 84.7 | 87.4 | 42.50 | <0.001 |
| Walk more | 88.8 | 86.9 | 90.1 | 79.6 | 83.2 | 85.7 | 75.73 | <0.001 |
| Avoid sugar sweetened soft drinks like Coca Cola | 81.5 | 70.9 | 81.5 | 79.9 | 60.9 | 74.8 | 157.20 | <0.001 |
| Eat smaller portions of food. | 85.2 | 61.3 | 77.0 | 74.4 | 63.5 | 72.1 | 190.94 | <0.001 |
| Eat breakfast every day | 76.3 | 79.7 | 57.7 | 68.6 | 74.8 | 71.5 | 158.78 | <0.001 |
| Try not to eat sweetened foods like cakes or confectionery | 75.7 | 67.9 | 68.8 | 69.9 | 64.4 | 69.3 | 39.05 | <0.001 |
| Eat foods with protein (e.g., meats, fish, eggs) every day | 72.4 | 70.8 | 70.2 | 64.5 | 57.3 | 67.0 | 112.80 | <0.001 |
| Avoid alcoholic drinks | 58.1 | 49.4 | 77.7 | 59.9 | 56.4 | 60.3 | 167.32 | <0.001 |
| Make a regular shopping list | 65.1 | 57.9 | 54.8 | 50.6 | 61.4 | 58.0 | 68.643 | <0.001 |
| Count the calories you consume | 49.2 | 57.1 | 66.1 | 48.4 | 67.8 | 57.8 | 113.73 | <0.001 |
| Don’t have second helpings | 72.4 | 58.9 | 66.1 | 59.1 | 31.2 | 57.3 | 516.02 | <0.001 |
| Don’t eat between meals | 46.0 | 58.4 | 68.7 | 48.0 | 53.8 | 55.1 | 121.26 | <0.001 |
| Try to eat less | 70.5 | 51.1 | 54.3 | 58.2 | 32.5 | 53.1 | 429.20 | <0.001 |
| Weigh yourself regularly | 39.0 | 47.5 | 56.7 | 52.0 | 53.8 | 49.9 | 87.13 | <0.001 |
| Join organized weight-loss programs like Weight Watchers | 47.6 | NA | 52.5 | 39.4 | 59.1 | 49.8 | 75.19 | <0.001 |
| Use smaller plates and dishes | 68.9 | 55.8 | 36.8 | 54.6 | 30.1 | 49.0 | 470.61 | <0.001 |
| Don’t sit down for longer than 15–20 min at a time | 43.8 | 68.9 | 40.0 | 42.0 | 47.7 | 48.6 | 222.85 | <0.001 |
| Don’t eat in front of the TV or computer | 40.2 | 58.4 | 32.9 | 42.5 | 51.6 | 45.2 | 189.12 | <0.001 |
| Go on a slimming diet | 30.3 | 41.0 | 50.4 | 30.9 | 57.3 | 42.2 | 202.49 | <0.001 |
| Substitute diet soft drinks for regular soft drinks | 23.5 | 31.7 | 46.8 | 29.3 | 62.6 | 39.0 | 424.30 | <0.001 |
| Use commercial meal replacements | 19.0 | 31.2 | 29.6 | 21.9 | 30.5 | 26.5 | 138.52 | <0.001 |
| Eat only with other people | 18.1 | 27.5 | 12.6 | 19.6 | 19.1 | 19.4 | 168.55 | <0.001 |
| Take diet pills | 10.0 | 20.3 | 17.4 | 16.6 | 20.4 | 17.0 | 75.17 | <0.001 |

**Table S3.** Adaption of Portrait of Values [1]. Your Personal Values (Guiding Principles in Your Life). How well do the following statements ACTUALLY describe you and your approach to life? Scale: 5 point: Not like me at all, Not like me, A little like me, Like me, Very much like me.

|  | **Melbourne  *n* = 769** | **Shanghai  *n* = 807** | **Indonesia  *n* = 788** | **Singapore  *n* = 771** | **Vietnam  *n* = 810** | **Total  *n* = 3945** | **Chi Sq** | ***p*** |
| --- | --- | --- | --- | --- | --- | --- | --- | --- |
| I believe everyone should have equal opportunities in life no matter who, where, or what they are. | 76.6 | 74.1 | 81.7 | 68.7 | 76.2 | 75.5 | 38.789 | <0.001 |
| I believe it’s important that every person in the world should be treated equally no matter who, where, or what they are. | 73.5 | 70.8 | 81.0 | 66.1 | 73.1 | 72.9 | 58.703 | <0.001 |
| I am a loyal friend and devoted to the people close to me. | 79.1 | 67.0 | 66.9 | 64.6 | 75.9 | 70.7 | 66.575 | <0.001 |
| I prefer to live in secure surroundings and avoid doing things that might endanger my safety. | 61.6 | 63.3 | 69.4 | 59.1 | 71.5 | 65.1 | 47.675 | <0.001 |
| I always try to be humble and modest and not draw attention to myself. | 61.8 | 57.4 | 77.3 | 62.0 | 66.0 | 64.9 | 109.460 | <0.001 |
| I am always willing to listen to people who are different and even when I disagree with them I still want to understand them. | 62.8 | 59.9 | 71.6 | 55.0 | 71.4 | 64.2 | 76.103 | <0.001 |
| I am free to make my own decisions about what I do and I’m not dependant on others. | 64.9 | 65.6 | 67.3 | 60.1 | 63.0 | 64.2 | 51.175 | <0.001 |
| I always try to help people around me and to care for their well-being. | 70.2 | 54.4 | 67.1 | 57.3 | 71.4 | 64.1 | 89.327 | <0.001 |
| I care for nature and always look after the environment. | 59.8 | 71.6 | 71.8 | 54.6 | 58.1 | 63.3 | 106.847 | <0.001 |
| I like to do things on my own | 55.8 | 61.8 | 69.7 | 60.6 | 68.5 | 63.3 | 55.037 | <0.001 |
| I believe that the government needs to be strong and ensure my safety against all threats | 58.8 | 71.9 | 50.0 | 61.9 | 64.0 | 61.4 | 169.937 | <0.001 |
| I always try to behave properly and to avoid doing anything people would say is wrong. | 57.1 | 44.1 | 61.8 | 52.0 | 68.9 | 56.8 | 135.205 | <0.001 |
| I always think up new ideas, be original and creative, and do things my own way. | 44.2 | 56.3 | 70.4 | 50.6 | 59.1 | 56.2 | 142.443 | <0.001 |
| I always try to follow the rules of society and do what is expected of me, even when no one is watching. | 54.7 | 54.4 | 61.8 | 48.6 | 56.2 | 55.2 | 36.094 | <0.001 |
| I adhere to traditions and try to follow the customs handed down to me by religion or family. | 43.6 | 58.5 | 64.5 | 44.0 | 53.1 | 52.8 | 163.161 | <0.001 |
| I take every chance I have to seek out fun and to always do the things that give me pleasure. | 35.1 | 63.9 | 47.6 | 42.0 | 70.4 | 52.1 | 334.107 | <0.001 |
| I like to be constantly surprised, to do many different things in my life, and always look for new things to do. | 39.0 | 48.2 | 54.9 | 44.0 | 52.3 | 47.8 | 73.281 | <0.001 |
| I always like to show my abilities and to be admired for what I do. | 37.2 | 59.1 | 46.8 | 41.8 | 47.3 | 46.6 | 110.440 | <0.001 |
| I always try to get respect from others and to get them to do as I say. | 33.9 | 55.1 | 22.1 | 44.2 | 64.4 | 44.2 | 673.461 | <0.001 |
| I always try to have a good time and to spoil myself | 30.4 | 30.1 | 57.6 | 41.8 | 59.8 | 44.0 | 411.864 | <0.001 |
| I like being seen as very successful and recognised by others for my achievements. | 27.3 | 59.2 | 36.9 | 43.1 | 48.4 | 43.2 | 311.529 | <0.001 |
| I always seek adventure and take risks to lead an exciting life. | 25.1 | 30.2 | 41.9 | 36.3 | 39.0 | 34.6 | 151.912 | <0.001 |
| I feel it’s important to be rich and have a lot of money and expensive things. | 16.8 | 44.9 | 29.7 | 34.4 | 35.8 | 32.4 | 264.500 | <0.001 |

Structural Equation Model: Analytical Procedure

SPSS 22 ([SPSS, 2014](#_ENREF_28)) and Mplus 7.2 [2] were used for the data analyses. Structural equation modelling (SEM) was employed to test the hypotheses. The robust Maximum likelihood (MLR) estimation method which is robust to non-normality of the data was used in the present analyses. Model evaluations were examined by chi-square statistics and accompanying significance tests. Goodness-of-fit indices reported are the Standardized Root Mean Square Residual (SRMR), Root Mean Square Error of Approximation (RMSEA), Tucker-Lewis index (TLI), and Comparative fit index (CFI) ([Marsh, Kit-Tai, & Zhonglin, 2004](#_ENREF_16)). When the models were considered to fit the data well, the following criteria were met: χ^2^ probability *p* > 0.05, SRMR < 0.05, RMESA < 0.05, TLI > 0.95, and CFI > 0.95.

A single indicator latent variable SEM approach [3] was used in the current analyses. The scale scores were formed by parcelling the items [4] ( for the personal values including security-conformity, equality, and exciting life; perceptions of the main cause of obesity including unhealthy behaviour, beyond personal control, and environment factors; and effective actions that individuals can take to maintain a healthy body weight including establishing healthy habits, getting healthy tips, and dieting. Once composite variables had been computed through parcelling the items measuring the same construct, both the regression coefficients, which reflect the regression of each composite variable on its latent variable, and the measurement error variances associated with each composite variable can be calculated via the formulae proposed by Munck.^2^. Using Munck’s formula, regression coefficients can be derived from SD $\sqrt{\alpha}$ and error variances from SD2 (1 − α). Both fixed values were used for single indicator construct in the structural equation model.

References

1. Schwartz, S.H.; Melech, G.; Lehmann, A.; Burgess, S.; Harris, M. Extending the cross-cultural validity of the theory of basic human values with a different method of measurement*. J. Cross-Cultur. Psychol.* **2001**, *32*, 519–542.
2. Muthén, L.K.; Muthén, B.O. Mplus User's Guide. Available online: http://www.statmodel.com/ugexcerpts.shtml (accessed on 18 January 2017).
3. Munck, I.M.E. *Model Building in Comparative Education: Applications of the LISREL Method to Cross-National Survey Data*, 6th ed.; Almqvist & Wiksell: Stockholm, Sweden, 1979.
4. Nasser, F.; Takahashi, T. The effect of using item parcels on ad hoc goodness-of-fit indexes in confirmatory factor analysis: An example using Sarason's reactions to tests*.* *Appl. Meas. Educ.* **2003**, *16*, 75–97.
